# Best practices for high data-rate macromolecular crystallography (HDRMX)

**DOI:** 10.1063/1.5128498

**Published:** 2020-01-09

**Authors:** Herbert J. Bernstein, Lawrence C. Andrews, Jorge A. Diaz, Jean Jakoncic, Thu Nguyen, Nicholas K. Sauter, Alexei S. Soares, Justin Y. Wei, Maciej R. Wlodek, Mario A. Xerri

**Affiliations:** 1 Ronin Institute for Independent Scholarship, c/o NSLS-II Bldg 745, Brookhaven National Laboratory, Upton, New York 11973, USA; 2 Ronin Institute for Independent Scholarship, 9515 NE 137th St., Kirkland, Washington 98034, USA; 3 Ronin Institute for Independent Scholarship, c/o NSLS-II Bldg 745, Brookhaven National Laboratory, Upton, New York 11973, USA; 4 Brookhaven National Laboratory, NSLS-II Bldg 745, Upton, New York 11973, USA; 5 Stony Brook University, Stony Brook, New York 11794, USA; 6 Lawrence Berkeley National Laboratory, 1 Cyclotron Rd., Berkeley, California 94720, USA; 7 Mount Sinai High School, 110 N Country Rd., Mt Sinai, New York 11766, USA

## Abstract

In macromolecular crystallography, higher flux, smaller beams, and faster detectors open the door to experiments with very large numbers of very small samples that can reveal polymorphs and dynamics but require re-engineering of approaches to the clustering of images both at synchrotrons and XFELs (X-ray free electron lasers). The need for the management of orders of magnitude more images and limitations of file systems favor a transition from simple one-file-per-image systems such as CBF to image container systems such as HDF5. This further increases the load on computers and networks and requires a re-examination of the presentation of metadata. In this paper, we discuss three important components of this problem—improved approaches to the clustering of images to better support experiments on polymorphs and dynamics, recent and upcoming changes in metadata for Eiger images, and software to rapidly validate images in the revised Eiger format.

## I. INTRODUCTION

Enabled by changes in technology, macromolecular crystallography is increasingly able to extend its focus from the average state observed in a single crystal or in a few merged crystals to studies of families of distinct structural states observed by single-shot or small wedge probes of a large ensemble of tiny crystals or microfocus probes of one or more larger crystals. This transition is driving a series of disruptive changes in the way diffraction data are collected, processed, and archived. Hardware improvements, such as fast high resolution detectors, high brilliance x-ray microbeams, and automated sample handling, are generating high data-rate and high data-volume data streams that conventional software packages and pipelines designed for simple single crystal experiments and one-node serial processing are not able to support or even keep up with. Past practice must be re-examined to ensure the quality of results and timely delivery. Networks and computational resources have had to be upgraded. Bottlenecks in pipelines must be removed, often by converting serial execution to parallel execution on multiple nodes, but those changes themselves can generate yet more network and computational load. Higher flux, smaller beams, and faster detectors open the door to experiments with very large numbers of very small samples that can reveal polymorphs and dynamics but require re-engineering of approaches to the clustering of images both at synchrotrons and XFELs (X-ray free electron lasers). The management of orders of magnitude more images and limitations of file systems favor a transition from simple one-file-per-image systems such as CBF to image container systems such as HDF5. This further increases the load on computers and networks, and the use of data coming from multiple runs at multiple beamlines requires a re-examination of the presentation of metadata. Over the past few years, recognition in the high data rate macromolecular crystallography community of the importance of complete and consistent metadata has grown. Such metadata is needed so that datasets can be easily processed at any site from data collected at different times, at another facility, or at multiple facilities, or months and years in the past.

Miller *et al.* (2019) and Basu *et al.* (2019) provided recent snapshots of the problem, some of the data collection strategies, and appropriate references.

In this paper, we discuss three important components of this problem: improved approaches to the clustering of images to better support experiments on polymorphs and dynamics, recent and upcoming changes in metadata for Eiger images, and software to rapidly validate images in the revised Eiger format.

## II. CLUSTERING OF IMAGES

**FIG. 1.**
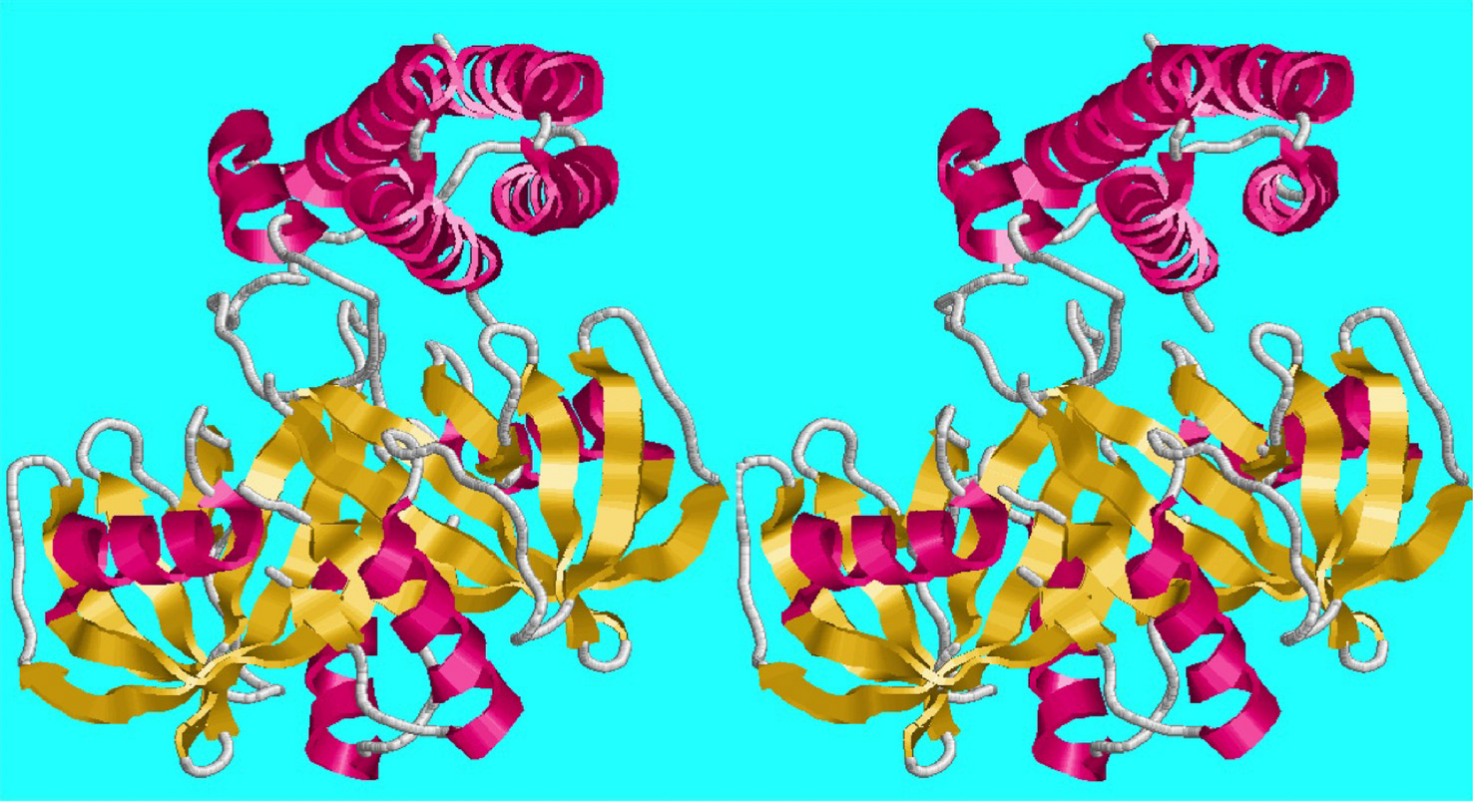
6NCH–crystal structure of CDP-Chase: multicrystal raster data collection (Miller *et al.,* 2019) structure from the PDB (Bernstein *et al.*, 1977) (Berman *et al.*, 2000). CPD-Chase is a CDP-choline pyrophosphatase. CDP-choline is Cytidine 5’-diphosphocholine.

**FIG. 2.**
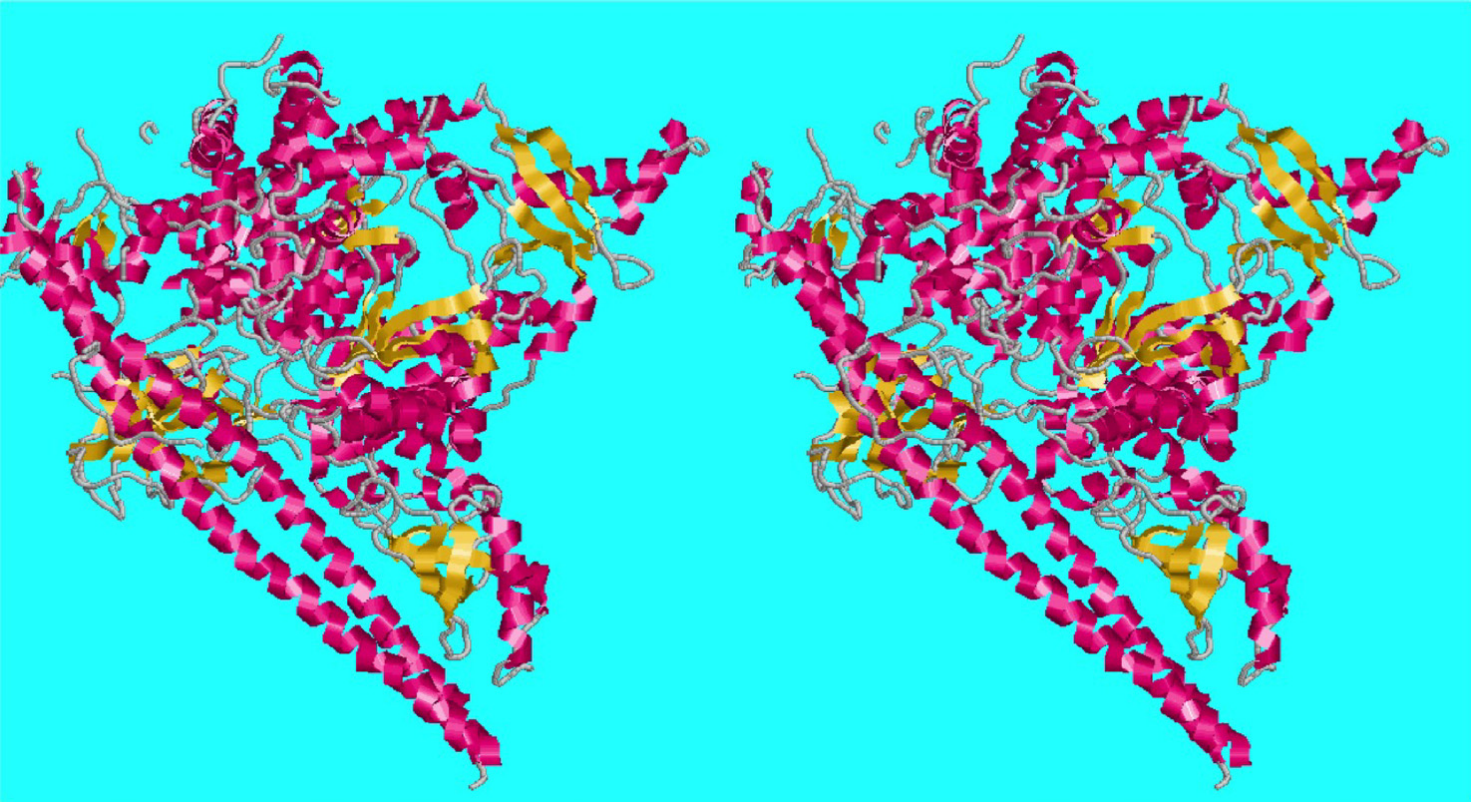
6NCT–structure of the p110alpha/niSH2-vector data collection (Miller *et al.,* 2019) structure from the PDB.

The serial crystallography of large numbers of small crystals or of multiple domains in larger crystals helps in coping with radiation damage and providing a sufficient number of images to probe multiple states and dynamics. See Fig. 1, a multicrystal raster-scan dataset, and Fig. 2, a vector-scan dataset, for examples of the structures produced in Miller *et al.* (2019) for a raster scan and a vector scan, respectively.

The diffraction images produced often are heterogeneous, of differing quality, ranging from no diffraction, to images with bad backgrounds, to single images with large portions of a clean reciprocal lattice, to images with spots from multiple lattices, to images whose meaning is very unclear. Clustering is the sorting of diffraction images into coherent sets of images that can be usefully processed together.

At Both XFELs and synchrotrons, successful serial crystallography depends on appropriate choices of clustering algorithms to segregate good images from bad and to sort into groups of closely related images (Foadi *et al.,* 2013; White *et al.,* 2012; Yamashita *et al.*, 2018; Zeldin *et al.,* 2015).

### A. Clustering practice

Clustering depends first on deciding what quanta of images are to be compared and merged: single frames (stills) or wedges. XFEL use favors stills. Stills can be created at synchrotrons. However, small wedges (say 5°) provide fewer partial spots, easier indexing, and more completeness. The wedges should not be so large that too many species would be averaged together and interesting polymorphs and dynamics would be lost.

Then, we decide what criteria will be used to discriminate among quanta of images: backgrounds, spot counts, lattices, and reflection intensities. If all that could be done by clustering were to allow us to segregate good images from bad, it would be well worth the effort. The move to microfocus beams and/or to microcrystals increases the chances that images will capture possible distinct states because fewer states will be averaged together in each image.

Backgrounds and spot counts are useful in discarding “bad” images.

Lattices and lists of reflections allow fine discrimination by distance, which opens up the possibilities of hierarchical and k-means clustering. Lattices are sensitive to changes in the gross morphology. Reflections are sensitive to changes in the range of resolutions compared. For reflections, the Pearson correlation coefficient (CC) can be used to compare reasonably complete sets of reflections.

Simple clustering may be done by the observation of graphs of selected variables in the data.

Clustering may be done hierarchically, making a dendrogram. In this approach, one lattice at a time is added to an evolving tree (dendrogram) of lattices until all the lattices have been gathered into one overall cluster. By slicing the tree at some fixed level, smaller, purer clusters are exposed.

Clustering may be done using *k*-means. In this approach, one starts with a guess, *k*, at the number of clusters and *k* starting cluster seed lattices. The remaining lattices are then added to whichever partially completed cluster is closest. This process may need to be restarted if the original seed lattices are unfortunately chosen.

The two approaches can interact. A dendrogram slice at an appropriately chosen level can provide suitable cluster centroids to use as *k*-means seed lattices.

### B. Issues in clustering

Some of the criteria used are simple, one-dimensional quantities, such as the number of spots found. When we are doing Bragg scattering experiments, we can use spot counts as a simple quality indicator for an image and put all the images with very few spots into a “discard” cluster.

However, when we are using lattice parameters as our criteria, the decisions are more complex. Having six lattice parameters, namely, [a,b,c,α,β,γ], does not mean we can, or should, apply the clustering techniques we would apply to data points in a six-dimensional vector space, where we can compute means and variances of data point coordinates and try to pick out the highest means with the smallest variances as our “best” clusters. Not only is it possible that the most interesting biology may be associated with some low-populated peaks, but it may also not even be meaningful to calculate a mean by averaging. For the reasons discussed by Andrews *et al.* (1980), Andrews and Bernstein (1988), (2014), and Andrews *et al.* (2019a), and (2019b), two lattices that seem to be far apart in terms of [a,b,c,α,β,γ] may actually be very close to each other when viewed in a different space. In order to cluster such lattices, we need to move away from the powerful tools provided when doing statistics in linear spaces and drop back to the cruder techniques needed to cluster in metric spaces, restricting our attention to working from the distances between lattices.

This does not mean we cannot use statistics to cluster, but it means we need to be creative and find a way to discover the dimension of a space without having been told it in advance, find the best cluster representative without having been able to calculate an average, and compute a measure of the variance of a cluster without actually being able to compute a variance by the usual formulas.

The minimal dimension of the space needed to represent a set of lattices tells us how many independent parameters really are needed to represent the data. The Hausdorff dimension (https://en.wikipedia.org/wiki/Hausdorff_dimension) at a point in a metric space can be determined by assuming the volumes of balls around that point go as a power of the diameters of the balls. Computationally, we find the power as the slope of the log of the volumes as a function of the diameter (Bernstein and Andrews, 2016). We substitute a count of the number of lattices found for the volume.

More problematic is designating a centroid when averages are not feasible. Given the dimension *N*, we can substitute one of the members of the set of lattices under consideration, but that requires us to find a lattice that is “central” in the set, rather than peripheral. We can do that by first picking a random lattice, *l_start_* in the set, and then *N* lattices $\left\{l_{n}|1\leq n\leq N\right\}$ such that *l*_1_ is as far as possible from *l_start_*, *l*_2_ is as far as possible from *l_start_* and *l*_1_ as possible, etc., and then picking a point to represent the set that minimizes the sum of the distances to $\left\{l_{n}|1\leq n\leq N\right\}$.

### C. Why cluster with better metrics

**FIG. 3.**
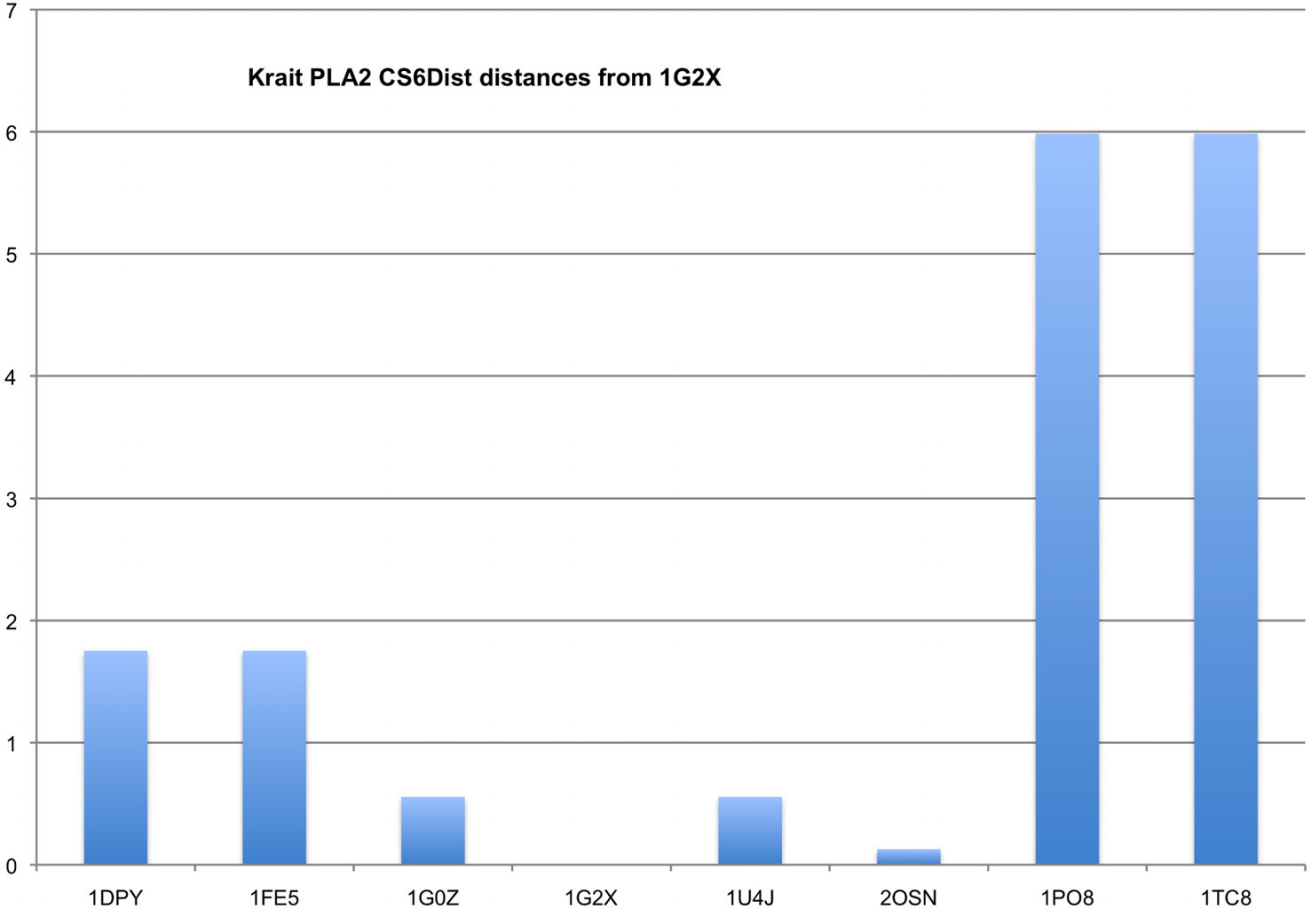
CS6Dist distances from 1G2X for krait Phospholipase A2 structures in the PDB as of September 1, 2019. The distances suggest two clusters, one with 1DPY, 1FE5, 1G0Z, 1G2X, 1U4J, and 2OSN and one with 1PO8 and 1TC8.

Le Trong and Stenkamp (2007) showed that there can be significant ambiguity in the association of particular space groups with crystallographic structures. There are eight X-ray structures for krait (*Bungarus caeruleus*) Phospholipase A2 in the PDB (Protein Data Bank) presented in several different space groups, six of which (1DPY, 1FE5, 1G0Z, 1G2X, 1U4J, and 2OSN) are structurally homologous as measured by FATCAT (Ye and Godzik, 2004), which compares backbone $C_{\alpha }$ positions, possibly with hinges introduced if needed. (We use the term “structurally homologous” in the sense of (Rossmann and Argos, 1976).] The other two (1PO8 and 1TC8) are structurally distinct and 6 Å from the others (Fig. 3). Le Trong and Stenkamp (2007) showed that “The two structures (1U4J and 1G2X) reported in space groups R 3 and C 2 are isomorphous with a third isoform with space group R 3 2 (1FE5). The original structure reports were interpreted in terms of different oligomeric forms of the isoforms, but these conclusions are not supported by the isomorphous structures.”

The six homologous structures currently in the PDB are listed in Table I. 1G2X is the approximate centroid of the cluster of 6. The distances from 1G2X are shown in Fig. 3.

**TABLE I.** Six structurally homologous structures from the eight krait (*Bungarus caeruleus*) Phospholipase A2 in the PDB.

PDB	Cell	Space Group (SG)	Primitive reduced cell
1DPY	(57.98, 57.98, 57.98, 92.02, 92.02, 92.02)	R 3 2	(57.98, 57.98, 57.98, 92.02, 92.02, 92.02)
1FE5	(57.98, 57.98, 57.98, 92.02, 92.02, 92.02)	R 3 2	(57.98, 57.98, 57.98, 92.02, 92.02, 92.02)
1G0Z	(80.36, 80.36, 99.44, 90, 90, 120)	H 3	(57.02, 57.02, 57.02, 89.605, 89.605, 89.605)
1G2X	(80.95, 80.57, 57.1, 90, 90.35, 90)	C 1 2 1	(57.098, 57.1065, 57.1065, 89.7325, 89.7519, 89.7519)
1U4J	(80.36, 80.36, 99.44, 90, 90, 120)	H 3	(57.02, 57.02, 57.02, 89.605, 89.605, 89.605)
2OSN	(57.1,57.1, 57.1, 89.75, 89.75, 89.75)	R 3 2	(57.104, 57.104, 57.104, 89.75, 89.75, 89.75)

If we were to prefilter clusters on the space group, we would organize these six entries into three separate clusters: 1DPY, 1FE5, and 2OSN in R 3 2, 1G0Z, and 1U4J in H 3, and 1G2X in C 1 2 1. However, if we start our clustering in P 1 by using the primitive reduced cells, we can explore the differences among all six entries together.

**Table:** 

*S*^6^ distance	1DPY	1FE5	1G0Z	1G2X	1U4J	2OSN
1DPY	0	0	1.720	1.753	1.720	1.757
1FE5	0	0	1.720	1.753	1.720	1.757
1G0Z	1.720	1.720	0	0.555	0	0.564
1G2X	1.753	1.753	0.555	0	0.555	0.127
1U4J	1.720	1.720	0	0.555	0	0.564
2OSN	1.757	1.757	0.564	0.127	0.564	0

If we use BGAOL (Andrews and Bernstein, 2014), which finds the Bravais lattice of the highest symmetry consistent with the submitted cell, on each of the primitive reduced cells, we see that all but one of them is actually equivalent to a *hR* cell, and the one that is not 1G2X is only 0.405 Å from an *hR*.

**Table:** 

PDB	Closest *hR* cell	Distance
1DPY	(83.429, 83.429, 96.820, 90, 90, 120)	0.0
1FE5	(83.429, 83.429, 96.820, 90, 90, 120)	0.0
1G0Z	(80.360, 80.360, 99.440, 90, 90, 120)	0.0
1G2X	(80.577, 80.577, 99.345, 90, 90, 120)	0.405
1U4J	(80.360, 80.360, 99.440, 90, 90, 120)	0.0
2OSN	(80.581, 80.581, 99.338, 90, 90, 120)	0.0

All the A chains are very similar to 117–118 residues aligned with a small RMSD (root mean square deviation) of aligned $C_{\alpha }$ atoms. The asterisks mark cases in which the sequences are identical. Note that even though 1G2X is in space group C 1 2 1, it really should be clustered with 2OSN in R 3 2.

**Table:** 

RMSD	1DPY	1FE5	1G0Z	1G2X	1U4J	2OSN
1DPY	0	0.13	0.47	0.43	0.48	0.43
1FE5	0.13	0	0.48	0.44	0.5	0.44
1G0Z	0.47	0.48	0	0.23	0.07*	0.22
1G2X	0.43	0.44	0.23	0	0.23	0.13*
1U4J	0.48	0.5	0.07*	0.23	0	0.23
2OSN	0.43	0.44	0.22	0.13*	0.23	0

**FIG. 4.**
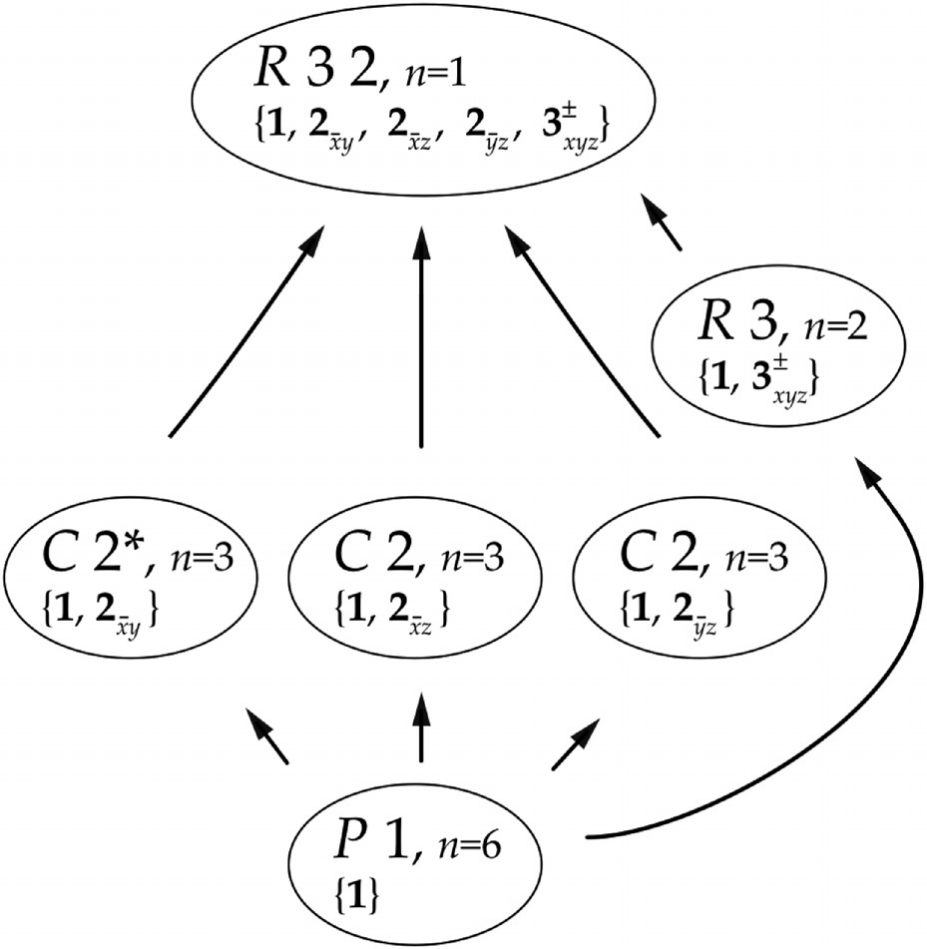
Relationships between alternate descriptions of the 1G2X cell.

The relationships between the alternate descriptions of the 1G2X cell are shown in Fig. 4. Although the structure is published in space group C 2 (noted with *), small perturbations of the atomic positions (averaging 0.16 Å, smaller than the coordinate uncertainty) can align this structure with a perfect R 3 2 lattice (Poon *et al.,* 2010). R 3 2 has five rotational subgroups (R 3, three C 2 settings, and P 1), each of which is an equally valid mathematical description. The group/subgroup relationships are depicted by arrows, and each group is listed with its rotational operators (1, identity; $2_{\bar{x}y}$, twofold rotation about [−1, 1, 0]; $2_{\bar{x}z}$, twofold rotation about [−1, 0, 1]; $2_{\bar{y}z}$, twofold rotation about [0, −1, 1]; and $3_{\mathrm{xyz}}^{\pm }$, forward and reverse threefold rotations about [1, 1, 1]). *n* refers to the number of phospholipase A2 polypeptide chains in the asymmetric unit, which are notionally related by noncrystallographic symmetry. In single-crystal data processing, available programs normally permit the selection of the highest symmetry that is consistent with the data. This analysis may be done at the level of Bravais lattice symmetry through the metric analysis of the unit cell parameters (Sauter *et al.*, 2004), Laue symmetry during the merging of symmetry-equivalent reflection intensities (Sauter *et al.*, 2006; Evans, 2006), or space group symmetry, upon the validation of the refined polypeptide structure (Poon *et al.,* 2010). For serial crystallography, the analysis of reflection intensities (Diederichs, 2017; Gildea and Winter, 2018) and unit cell clusters warrants a global analysis of all the data.

The major lessons of this example are to cluster with metrics that can work across space groups and to change the clustering pipelines not to depend on the initial selection of a single space group because images from different space groups may be surprisingly close to one another, especially for room temperature work and for polymorphs and dynamics, or the space group simply may be misidentified. To avoid getting trapped into averaging incommensurate cases, stop building lattice clusters at minimally acceptable completeness. To go further, cluster on other criteria such as correlation coefficients among sets of reflections (CC).

## III. CHANGES IN METADATA

Since the introduction of the CBF format (Bernstein and Hammersley, 2005) for the Dectris Pilatus detectors in 2007, there has been a recognition of the importance of controlling the metadata associated with images in order to both ensure that essential information is not lost and minimize delays in handling the metadata. When the Eiger detectors were introduced, the community agreed to adopt the NeXus/HDF5 format for efficiency in handling the much larger volume of data with fewer files to reduce file system and network burdens. Much of the metadata is carefully aligned between NeXus/HDF5 and CBF under an agreement between the NeXus International Advisory Committee (NIAC) and the IUCr Committee for the Maintenance of the CIF Standard (COMCIFS). With the co-operation of Dectris, the high data rate macromolecular crystallography (HDRMX) group and website were established to facilitate the community discussion of the software, data, and metadata.

### A. HDRMX metadata discussions

There are signs of divergence among beamlines in Eiger formats, and it is time to add new metadata, for example, to identify beamlines and facilities and to record metadata that will be helpful in PDB depositions. The primary objective is to ensure that sufficient metadata will be provided to allow processing at a facility other than the one at which the data were produced. In particular, detailed descriptions of axis chains to be used to process the data are needed, for both sample goniometers and detector positioners. The HDRMX group meets frequently at conferences and conducts internet discussions as well. The HDRMX group has come to the conclusion that complete and consistent metadata sufficient to allow data collected at one beamline to be processed at other times and at other locations than where and when it was originally collected is important and is proposing a new “Gold Standard” for macromolecular crystallography data collected at light sources.

### B. Structure of the new metadata

In general, the requested augmentation of metadata is divided into two groups: first, metadata to be added via a templating mechanism in the Dectris software to be configured before collection as static changes to the “master” files and, second, metadata to be added after collection, possibly via H5copy. For simplicity, we refer to the former as static and the latter as dynamic.

### C. Static metadata

Some tags for static (i.e., Dectris template) additions are already available. imgCIF defines AXIS tags needed for the specification of arbitrary and very general axis chains. NeXus defines the equivalent information in the NXtransformation base class.

Concern has been expressed about cluttering the templating mechanism with large numbers of tags used only in the most complex cases. To avoid such clutter, the input to the template can be the path to either a CBF or a NeXus file with the appropriate axis information, along with the necessary software to automatically convert between CBF and NeXus axis conventions. One way or another all diffraction geometry and all detector geometry need to be described. Tags have been defined to carry metadata specifying the beamline and facility. Note that the detector distance, wavelength, and beam center are already specified and very necessary. As integrating detectors or other detectors that do not count single photons come into use in this performance range, detector gain will need to be specified. Tags are needed for the HDF5 software version to declare the use of nonstandard local format conventions, to list the files comprising a dataset, and to give the format of each particular file.

As a partial example, consider a beamline called XXX (ID1) at site SYNC with an omega axis and pin_x, pin_y, and pin_z translation axes stacked 5 mm apart, using hdf5_1.8.14 and NXmx 1.4. Then, a portion of the necessary information presented as a CIF file might be as shown below

$$\begin{array}{l} \mathrm{data\_AMX\_metadata}\\ \;\;\mathrm{loop\_}\\ \;\;\mathrm{\_axis.id}\\ \;\;\mathrm{\_axis.type}\\ \;\;\mathrm{\_axis.equipment}\\ \;\;\;\;\mathrm{\_axis.depends\_on}\\ \;\;\;\;\;\mathrm{\_axis.vector[1]}\\ \;\;\;\;\;\;\mathrm{\_axis.vector[2]}\\ \;\;\;\;\;\;\;\mathrm{\_axis.vector[3]}\\ \;\;\;\;\;\;\;\;\mathrm{\_axis.offset[1]}\\ \;\;\;\;\;\;\;\;\;\mathrm{\_axis.offset[2]}\\ \;\;\;\;\;\;\;\;\;\;\mathrm{\_axis.offset[3]}\\ \;\;\mathrm{Source . source . 0 0 1 . . .}\\ \;\;\mathrm{Gravity . gravity . 0 – 1 0 . . .}\\ \;\;\mathrm{pin\_x translation goniometer . -1 0 0 0 0 0}\\ \;\;\mathrm{Omega rotation goniometer pin\_x 1 0 0 –5 0 0}\\ \;\;\mathrm{pin\_y rotation goniometer omega 0 1 0 –10 0 0}\\ \;\;\;\mathrm{pin\_z rotation goniometer pin\_y 0 0 –1 –15 0 0}\\ \;\;\mathrm{\_array\_intensities.gain 1.0 \#counts/photon}\\ \;\;\mathrm{\_diffrn\_source.source SYNCHROTRON}\\ \;\;\mathrm{\_diffrn\_source.type 'SYNC XXX (ID1)'}\\ \;\;\mathrm{\_diffrn\_source.pdbx\_synchrotron SYNC}\\ \;\;\mathrm{\_diffrn\_source.pdbx\_synchrotron\_beamline 'XXX (ID1)'}\\ \;\;\mathrm{\_dataset\_file\_format.file\_format 'hdf5\_1.8.14 and NXmx 1.4'}\\ \;\;\mathrm{\_diffrn\_radiation.beam\_width 7 ♯micrometres}\\ \;\;\mathrm{\_diffrn\_radiation.beam\_height 5 ♯micrometres}\\ \;\;\mathrm{\_diffrn\_radiation.beam\_flux 400000000000 ♯ph/s in the beam} \end{array}$$

There will be some conversions in mapping to the NXmx NeXus/HDF5 version. For example, the _diffrn_source.pdbx_synchrotron CIF tag value will be used to populate the NXmx

/entry:NXentry/source:NXsource/namefield and the _diffrn_source.pdbx_synchrotron_beamline CIF tag value will be used to populate the NXmx

/entry:NXentry/instrument:NXinstrument/name field.

### D. Dynamic metadata

Many tags for dynamic (non-Dectris-template) additions are also already available. For example, the monochrometer, the beam_height, beam_width, beam_flux, and sample sequence can all be placed by a beamline or user in a CIF or NeXus file for merging with H5copy into an existing master metadata file. The existing imgcif and mmcif dictionaries provide appropriate tags to use, and more can be added. The following have been discussed: sample provenance, sample physical characteristics, sample imagery, protein sequence, detector, and sample environments, including temperature, sample delivery method, serial crystallography parameters (including pump probes), spectroscopy, sample mount, detector ROI (region of interest), beamline optics, and source parameters, e.g., mode, current, collection strategy, scan type, scan mode, beam profile (Gaussian and tophat), monochromator bandpass, beam divergences, and beam collimation.

## IV. VALIDATE IMAGES

Especially with new metadata being added, a fast data-driven tool for NeXus/HDF5 image validation is needed. The best available tool is cnxvalidate by Mark Koennecke

https://github.com/nexusformat/cnxvalidate which is data driven, working against

https://github.com/nexusformat/definitions.

For development, we are maintaining a fork of the validator at

https://github.com/HDRMX/cnxvalidate.

For development, we are maintaining a fork of the definitions at

https://github.com/HDRMX/definitions.

Typical call and output are

**Table:** 

nxvalidate -a NXmx -l ~/definitions\
-e thau2_25dps_tr0p05_1_master.h5
message=“Missing required global file_name
attribute”
… sev=error dataPath=/dataFile
=thau2_25dps_tr0p05_1_master.h5

The necessary changes were agreed at the Diamond Light Source HDRMX meeting 6–7 November 2019. The agreed changes will be integrated with the development version of cnxvalidate and submitted to Dectris and NIAC for review shortly. They are available for consideration on the HDRMX web site http://hdrmx.medsbio.org.

If all goes well, users should start seeing validated gold standard images in use in early 2020.

## V. CLUSTERING BEST PRACTICE CONCLUSIONS

Best practice depends on the details of the experiment. For example, the choice of clustering by the space group vs structure factors may depend on whether conformational changes being sought are sufficiently external to distort cell edges or are buried.

In the presence of radiation damage, it is important to detect and discard bad images (no spots, or not indexed) first.

If the stills and wedges can be indexed, the next step is to index each still or wedge.

It is a common practice to sort by the resulting likely space group and then cluster on lattice parameters; this may be a mistake because, as the krait Phospholipase A2 example in Sec. II C shows, structures from different space groups may actually be structurally homologous.

In simple cases, it may be sufficient to do histograms or scatter plots on cell edge lengths and pick out the peaks; this may also be a mistake.

**FIG. 5.**
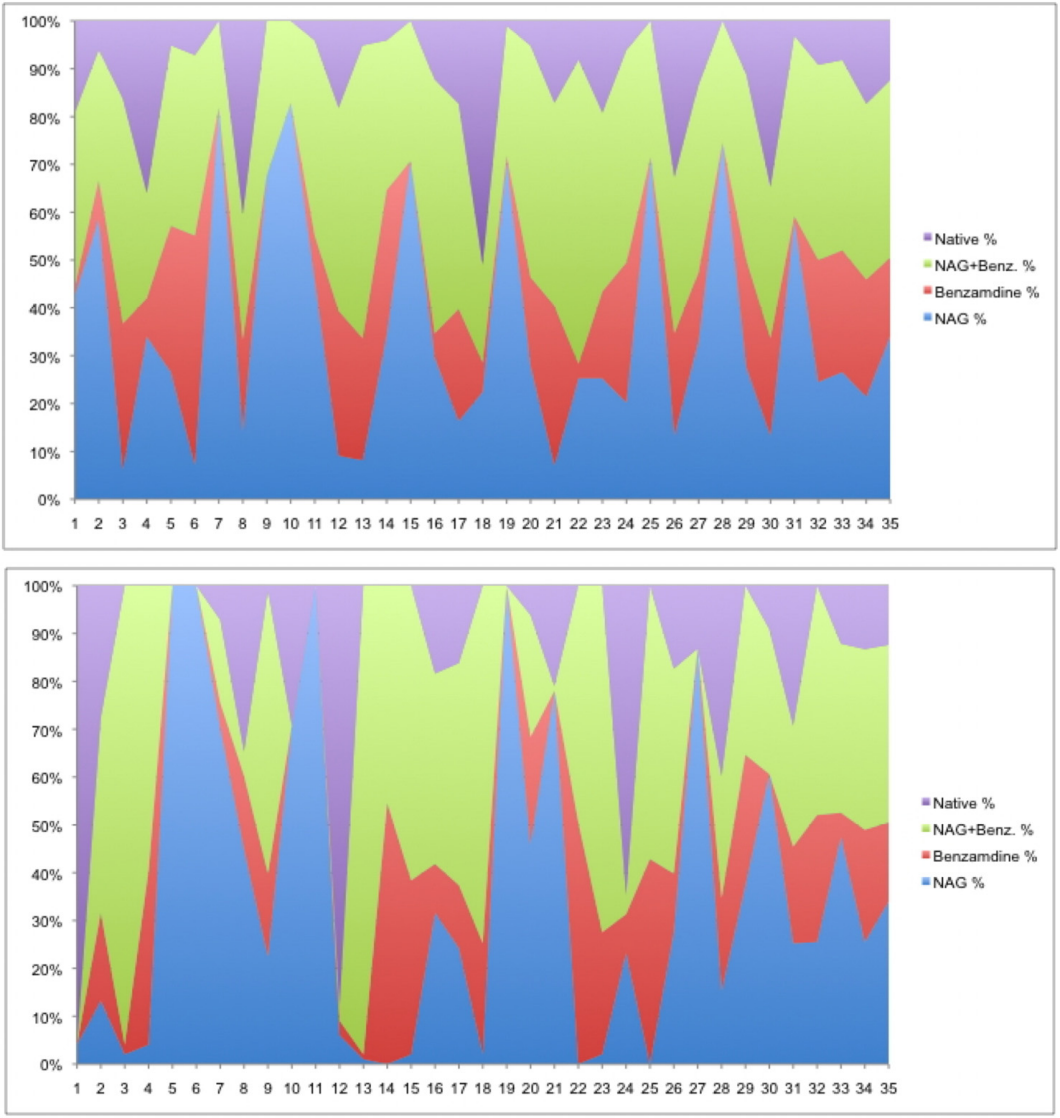
Purity of clusters in the NAG-benzamidine soak experiment (NAG is N-acetylglucosamine). When a color reaches from the bottom to the top, the cluster is purely that species. The image on the top is a lattice-only clustering. The image on the bottom is a lattice-first-structure-factor-second clustering for which the clusters are very pure. Reproduced with permission from Bernstein *et al.*, preprint bioRxiv:141770 (2017). Copyright 2017 Authors, licensed under a Creative Commons Attribution (CC-BY 4.0 International) license.

A reasonable process to consider is doing just enough lattice clustering on stills or wedges to be able to merge to a moderate degree of completeness and then doing reflection clustering on those lattice-merged clusters (Bernstein *et al.*, 2017). This can have a significant impact on the purity of the clusters obtained as shown in Fig. 5.

In a world of ever increasing data rates and datasets of thousands to hundreds of thousands of images from large numbers of crystals, consistency in metadata to allow for data collected at multiple times from multiple sites to be processed easily at different times and places from where the data were originally collected is increasingly important.
